# Effects of Metamizole, 4-Methylaminoantipyrine, and 4-Aminoantipyrine on LX-2 Liver Cell Line Viability and Apoptosis

**DOI:** 10.3390/molecules30010017

**Published:** 2024-12-24

**Authors:** Georgiana-Iulia Lupu, Emoke Pall, Mihai Cenariu, Monica Irina Nan, Sanda Andrei

**Affiliations:** 1 Department of Preclinical Sciences, Faculty of Veterinary Medicine, University of Agricultural Sciences and Veterinary Medicine Cluj-Napoca, 400372 Cluj-Napoca, Romania; georgiana-iulia.lupu@usamvcluj.ro (G.-I.L.); monica.nan@usamvcluj.ro (M.I.N.); 2 Department of Clinical Sciences, Faculty of Veterinary Medicine, University of Agricultural Sciences and veterinary Medicine Cluj-Napoca, 400372 Cluj-Napoca, Romania; emoke.pall@usamvcluj.ro (E.P.); mihai.cenariu@usamvcluj.ro (M.C.)

**Keywords:** metamizole, metabolites, hepatotoxicity, liver cell lines

## Abstract

Metamizole (dipyrone) is a non-opioid analgesic widely used in human and veterinary medicine, despite ongoing concerns about its safety due to risks such as agranulocytosis and potential hepatotoxicity. This study investigates the cytotoxic (MTT assay) and pro-apoptotic effects of metamizole and its primary metabolites, 4-methylaminoantipyrine (4-MAA) and 4-aminoantipyrine (4-AA), on the LX-2 liver cell line. These metabolites are implicated in both the therapeutic and adverse effects of the drug. The objective is to elucidate the mechanisms of potential hepatotoxicity, with a focus on cell viability and apoptosis. Metamizole was tested at five concentrations (100, 200, 400, 600, and 1000 µg/mL), while its metabolites were tested at two concentrations (100 and 1000 µg/mL). The results show a dose-dependent decrease in cell viability, with significant reductions at higher concentrations. The greatest cytotoxic effects were observed with 4-AA and 4-MAA, which induced marked apoptosis at 1000 µg/mL. This study concludes that metamizole and its metabolites can cause liver cell damage, underscoring the importance of caution in its clinical use and the need for further research to ensure its safety.

## 1. Introduction

Metamizole (dipyrone) is a widely used non-opioid analgesic in both human and animal medicine. Metamizole’s safety has been the topic of numerous debates, given the fact that in certain countries metamizole is still available by prescription, while in others, it is banned due to the risk of agranulocytosis [1].

Our study seeks to address a key gap in understanding the safety of metamizole when used as an analgesic in non-conventional species (NACs). Given the limited options available for effective and safe pain management in exotic animals, it is crucial to explore drugs like metamizole, which is already used in some veterinary clinics. Investigating its hepatotoxic potential provides essential data for developing safer analgesic protocols, ultimately improving the standard of care for these unique and sensitive patients.

Pain management in these species is often challenging, with many cases of pain being under-recognized or inadequately treated. Postoperative pain management is critical for their welfare, as untreated pain can lead to prolonged recovery times, a reduced quality of life, and other complications. Furthermore, small mammals such as rabbits, ferrets, rodents, and hedgehogs have become some of the most common pets present in veterinary practice, and each of these species have specific analgesic needs due to their anatomy and physiology [1].

Metamizole (MT) is an analgesic and antipyretic drug labeled for use in humans, horses, cattle, swine, and dogs. MT is rapidly hydrolyzed to the primary metabolite 4-methylaminoantipyrine (4-MAA) [2].

**Figure 1:**
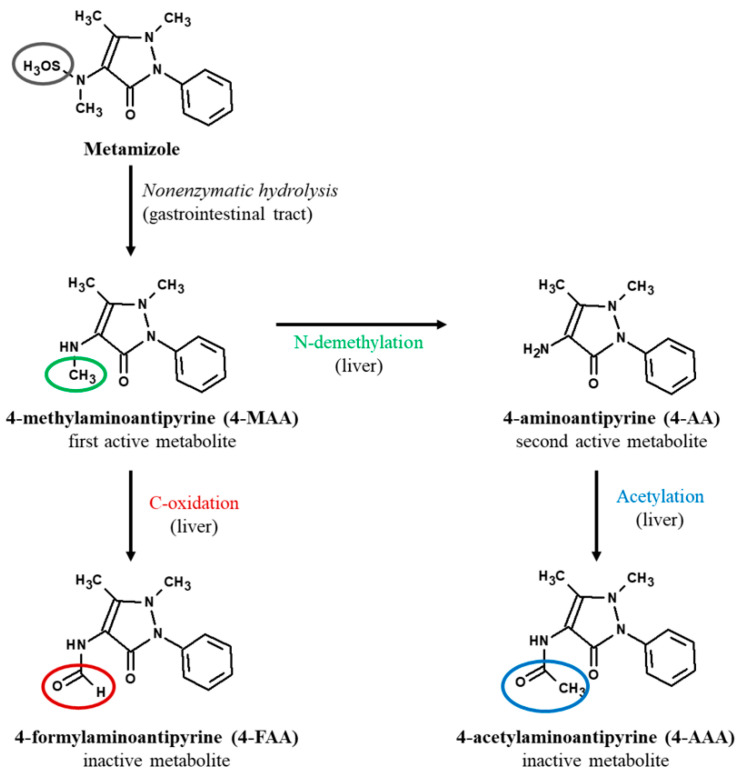
Hepatic metabolism of metamizole [1].

Metamizole is largely metabolized in the liver, and this action is mediated by the cytochrome P450 complex. Taking this into account, the hepatotoxic potential of this drug is easy to imagine [1,3]. Metamizole is considered a prodrug, which indicates that it metabolizes and is broken down in the body very rapidly—when it is administered orally—via a nonenzymatic hydrolysis reaction into its primary metabolite 4-methylaminoantipyrine (4-MAA). This form is completely absorbed in gastric juice and then transferred to the liver, where it is mediated by the cytochrome P450 (CYP) 3A4 system, which is essential for metabolic reactions. Further, 4-MAA undergoes two metabolic processes in the liver. The first reaction forms 4-aminoantipyrine (4-AA), which is a second active metabolite. The second reaction, a C-oxidation, forms a first inactive metabolite called 4-formylaminoantipyrine (4-FAA). In addition, when 4-AA is further acetylated, it then forms another inactive metabolite 4-acetylaminoantipyrine (4-AAA) (Figure 1) [1,3,4,5].

Metamizole’s use in human medicine is now controversial. From a veterinary medicine standpoint, the literature does not provide conclusive data on the efficacy of this substance, but we have, as a starting point, a variety of studies conducted on swine, cattle, canids, and equines, as well as a meta-analysis of the therapeutic effects of dipyrone on dogs conducted by Brazilian researchers [6].

Metamizole exhibits mild anti-inflammatory and antithrombotic properties. Its analgesic effects are primarily attributed to its metabolite, 4-methylaminoantipyrine, which inhibits the production of prostaglandin E2 (PGE2). The second metabolite, 4-aminoantipyrine, reduces PGE2-induced pain behaviors by desensitizing the TRPV1 (transient receptor potential cation channel subfamily V member 1) heat receptor through the activation of the cannabinoid CB1 receptor [7,8].

Previous studies have shown that metamizole is associated with a major risk of liver damage, but there is currently limited data available on the comparative risk of liver injury associated with metamizole. Through a comparative cohort analysis, researchers investigated incident users and found that while metamizole is widely used for its analgesic and antipyretic properties, its hepatotoxic risk, though present, remains lower or comparable to other commonly used pain medications, such as paracetamol [9]. However, these results should be interpreted cautiously, considering potential residual confounding and the need for corroborating data from other studies. Currently, there is little data in the literature on the potential hepatotoxicity of metamizole, and the aim of our study is to take this hypothesis further in order to find a drug with hardly any adverse reactions in veterinary medicine. As we know from previous studies [10], the risk of the prolonged use of metamizole in humans can lead to severe agranulocytosis, which is why it is banned in some countries (such as the United States, the United Kingdom, and Sweden). On the other hand, it is still accessible on the market as a prescription-only medicine in several countries, such as Brazil, Mexico, China, Switzerland, Germany, and Eastern Europe [1,3].

Although the drug in question has been on the market for nearly a century, the substance’s mode of action and toxicity mechanism are still not entirely known. Furthermore, the enzymes involved in metamizole metabolism remain mostly unexplored. Although it is emphasized that its use is safer compared to other analgesics, especially for the short-term treatment of pain, information on its safety in medium- and long-term use is limited [11].

The primary goal of this study was to investigate the cytotoxic and pro-apoptotic activity of metamizole (M) and its two primary metabolites, 4-methyl-amino-antipyrine (4-MAA) and 4-amino-antipyrine (4-AA), on liver cell line LX-2 in an environment where data is scarce and often contradictory. This study was driven by a number of theories suggesting that metamizole may have a negative effect on liver function and can cause liver damage through a direct toxic mechanism on cells.

Given the need for a reliable analgesic with minimal side effects, particularly in post-operative pain management, further research is essential to establish the safety profile of metamizole for clinical applications.

## 2. Results

### 2.1. Cell Viability

Cytotoxicity testing of metamizole and its metabolites was performed using the liver cell line LX-2. Hepatic stellate cells (HSCs) (formerly known as Ito cells, lipocytes, or fat storage cells) are a major cell type responsible for liver fibrosis following their activation into myofibroblast-like fibrogenic cells [12].

To evaluate the cytotoxic potential of metamizole, five concentrations of a metamizole solution (100, 200, 400, 600, and 1000 µg/mL) and two concentrations of its metabolites, 4-MAA and 4-AA (100 and 1000 µg/mL), were selected for this study.

According to the results of an MTT assay, metamizole and its metabolites can significantly reduce cell viability in a dose-dependent manner (*p* < 0.05). After 24 h of treatment with metamizole solutions, cell viability testing demonstrated a decrease in viability with increasing metamizole concentrations.

The control group consisted of untreated cells, which exhibited 100% viability. Viability results from cells treated with metamizole and its two metabolites were compared to this control group.

**Figure 2:**
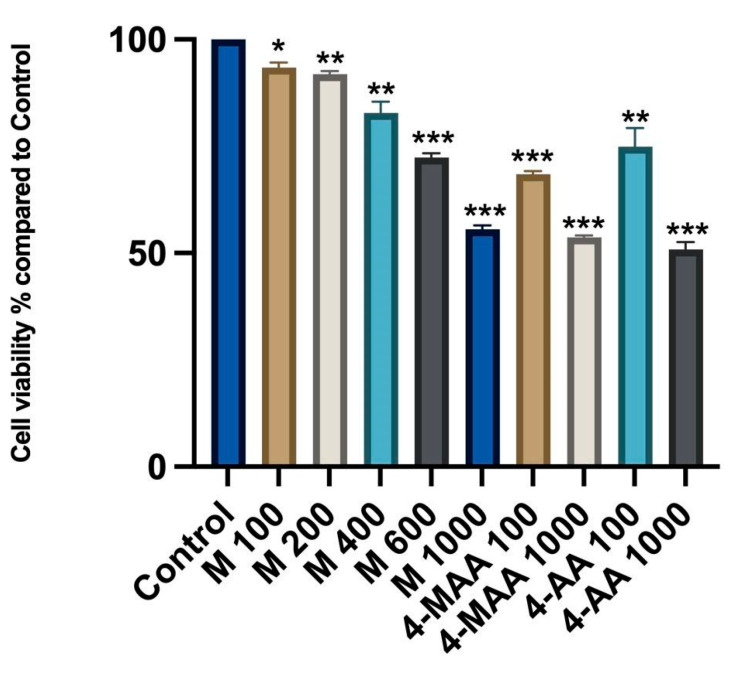
MTT test results. Viability of liver stellate cells (LX-2) are expressed in percentages (using *t*-test) and were treated with different concentrations (100, 200, 400, 600, and 1000 µg/mL; * *p* < 0.05 vs. control; ** *p* < 0.01 vs. control; *** *p* < 0.001 vs. control) of metamizole (M) and its two metabolites, 4-MAA and 4-AA (100 and 1000 µg/mL; ** *p* < 0.01 vs. control; *** *p* < 0.001 vs. control). The findings show the mean ± SD of the measurements made in triplicate.

Figure 2 illustrates the viability of liver stellate cells (LX-2), expressed as percentages, measured after treatment with different concentrations of metamizole (100, 200, 400, 600, 1000 µg/mL) and its two metabolites, 4-MAA and 4-AA (100 and 1000 µg/mL). The data were analyzed using a *t*-test to compare each treatment group to the control group. Statistically significant differences were observed (*p* < 0.05), indicating varying levels of significance for the effects of metamizole and its metabolites on cell viability. The findings, expressed as the mean ± SD of triplicate measurements, highlight the dose-dependent toxicity or cytotoxicity of the compounds tested, as determined by a *t*-test analysis.

At a concentration of 100 µg/mL, 4-MAA and 4-AA reduced cell viability to 68.4% and 74.86%, respectively, in contrast to the metamizole solution, which decreased cell viability to 93.39% at the same concentration.

As seen in Figure 2, cell viability gradually decreases at higher concentrations of metamizole, with a noticeable decrease at the highest concentration of metamizole (1000 µg/mL).

The lowest cell viability percentage of 50.86% was recorded at a concentration of 1000 µg/mL for the metabolite 4-AA, followed by the 53.63% recorded at 1000 µg/mL of 4-MAA (Figure 2).

**Figure 3:**
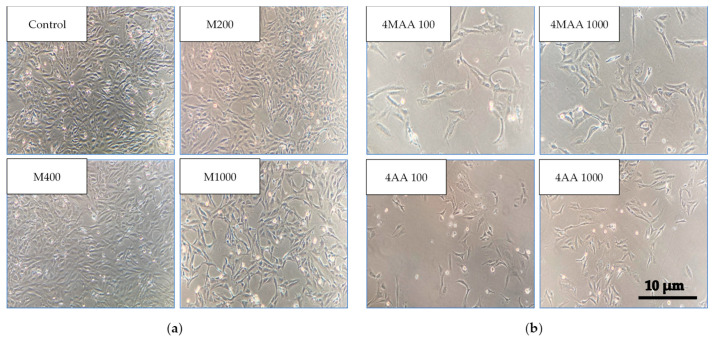
The appearance of LX-2 cells treated with different concentrations of metamizole and the two metabolites. (**a**) The appearance of control LX-2 cells before exposure and LX-2 cells treated with 200, 400, and 1000 µg/mL of metamizole solution (M). (**b**) The appearance of LX-2 cells treated with 100 and 1000 µg/mL of 4-methyl-amino-antipyrine (4-MAA) and 4-amino-antipyrine (4-AA).

The aforementioned aspects can also be confirmed by Figure 3. Therefore, it can be observed that the number and appearance of cells are strongly influenced and directly proportional to the concentration of metamizole. Exposure to the two metabolites results in more significant cellular thinning and morphological alterations.

### 2.2. Apoptosis

Control samples represent the condition of cells not treated with the metamizole solution. Cell quality can be affected by the culture medium used, as well as the conditions of cultivation and preservation.

**Figure 4:**
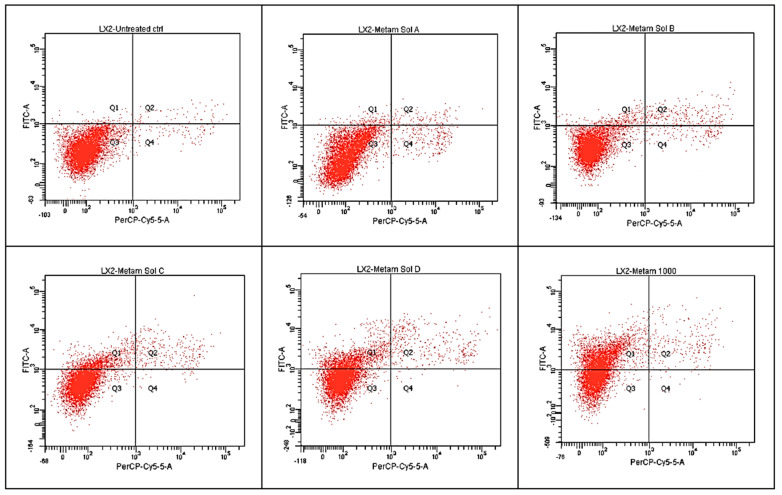
Analysis by flow cytometry of the viability of the untreated cells (control) and the viability of cells treated with a metamizole solution (M) at different concentrations (100, 200, 400, 600, and 1000 µg/mL); (Q1 = early apoptotic cells, Q2 = late apoptotic cells, Q3 = live cells, and Q4 = necrotic cells).

Details revealed by a flow cytometry examination of LX-2 hepatic cells can be observed in Figure 4. Using this method, we also found that exposure to the metamizole solution significantly influenced cell viability. In the control samples, 97.6% of the cells were viable, with 0.6% of the cells in early and late apoptosis and 1.1% of necrotic cells. For cells treated with the highest concentration of metamizole (1000 µg/mL), only 54.3% of them remained viable after exposure. The one in question contained 43% early apoptotic cells, 2.5% late apoptotic cells, and 0.2% necrotic cells.

**Figure 5:**
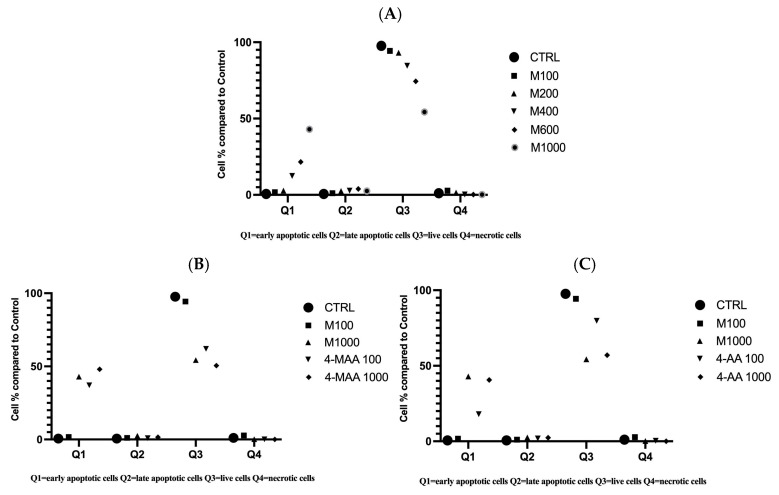
(**A**) Flow cytometry analysis of LX-2 cells following exposure to metamizole (M) solution at 5 different concentrations. (**B**) Flow cytometry analysis of LX-2 cells following exposure to metamizole (M) solution in comparison with 4-MAA at 2 different concentrations. (**C**) Flow cytometry analysis of LX-2 cells following exposure to metamizole (M) solution in comparison with 4-AA at 2 different concentrations (Q1 = early apoptotic cells, Q2 = late apoptotic cells, Q3 = live cells, and Q4 = necrotic cells).

**Figure 6:**
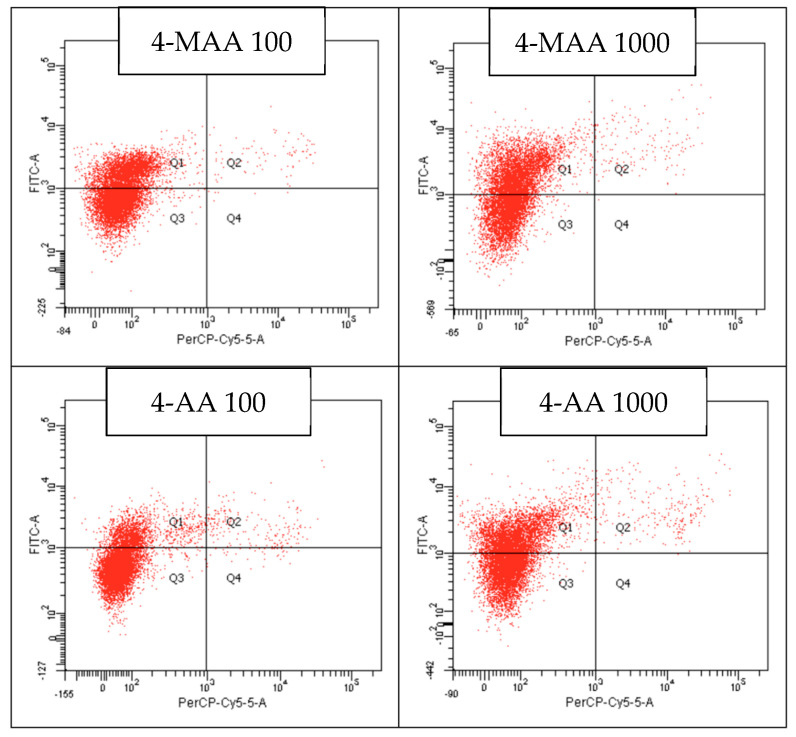
Analysis by flow cytometry of the viability of cells treated with the 2 metabolites of metamizole at the 2 concentrations (Q1 = early apoptotic cells, Q2 = late apoptotic cells, Q3 = live cells, and Q4 = necrotic cells).

At a concentration of 1000 µg/mL, 4-MAA reduced cell viability to 50.5%, with 48% of the cells in early apoptosis, 1.6% in late apoptosis, and 0% in necrosis. For the metabolite 4-AA, at a concentration of 1000 µg/mL, cellular viability decreased to 57%, with 40.6% of the cells in early apoptosis, 2.4% in late apoptosis, and 0% in necrosis. On the other hand, cellular viability at the lowest concentration of 100 µg/mL of the metamizole solution was 94.3%, with 1.7% of the cellular population in early apoptosis, 1.1% in late apoptosis, and 2.8% in necrosis. For the first metabolite, 4-MAA, at the same concentration of 100 µg/mL, 62% of the cells maintained their viability, with 37.1% in early apoptosis, 0.9% in late apoptosis, and 0.1% in necrosis. At the same concentration of 100 µg/mL of 4-AA, cellular viability was 79.8%, with 18% of the cells in early apoptosis, 1.9% in late apoptosis, and 0.4% necrotic cells. Similar to the MTT test, a decrease in the viable cell percentage was observed with an increase in the concentration of the metamizole solution (Figure 5 and Figure 6).

The highest percentage of necrotic cells (2.8%) was recorded at the lowest concentration of metamizole (100 µg/mL), followed by a percentage of 1.5% at a concentration of 200 µg/mL of metamizole. The percentage of necrotic cells decreases with an increase in the concentration of metamizole, a trend that can also be observed with the metabolites.

For the early apoptosis stage, the percentage of cells in this phase increases with concentration. The highest percentage (48%) was recorded for the metabolite 4-MAA at a concentration of 1000 µg/mL, followed by 43% at a concentration of 1000 µg/mL of metamizole.

For the late apoptosis stage, the percentage of affected cells is significantly lower, with the highest number being 3.9% at a concentration of 600 µg/mL of metamizole.

## 3. Discussion

The metabolites, 4-MAA and 4-AA, at their lowest concentration of 100 µg/mL, were more potent in decreasing cell viability compared to the lowest concentration of the metamizole solution. 4-MAA can be demethylated into 4-AA or formylated into N-formyl-4-aminoantipyrine (4-FAA). 4-AA can be acetylated to N-acetyl-4-aminoantipyrine (4-AAA), which is primarily excreted in urine. Only 3% of administered metamizole is excreted in urine as 4-MAA, with the rest being excreted mainly as 4-AA, 4-AAA, and 4-FAA, as well as other minor metabolites [13].

The second active metabolite of metamizole, 4-AA, has a longer pharmacological half-life but a significantly lower analgesic effect and a longer time to reach the maximum plasma concentration than 4-MAA. The rapid and consistent efficacy of metamizole is attributed to the combined characteristics of 4-MAA and 4-AA [5].

The half-life of 4-MAA is approximately 2.7 h [14,15], which corresponds to approximately 500 μmol/h. Since almost all 4-MAA is eliminated through demethylation, this rate can be compared to the hepatic microsomal capacity of 16.8 μmol/h, suggesting a high extrahepatic demethylation capacity for 4-MAA [13].

Therefore, both metabolites exhibit clinically relevant characteristics, such as a rapid onset and long duration of effects, allowing for dosing intervals of 6 to 8 h. However, the half-life of MAA is dose-dependent. The other two metabolites, FAA and AAA, are inactive. Certain metabolites that contribute to analgesic efficacy and severe side effects are still unknown [16].

In people with impaired liver function, the half-life of 4-MAA is prolonged, which supports the hypothesis that 4-MAA is metabolized by the liver [15,17].

After ingestion, metamizole is hydrolyzed in the gastrointestinal tract to 4-methylaminoantipyrine (MAA), which is rapidly and almost completely absorbed from the digestive system. MAA is further metabolized in the liver by demethylation to 4-aminoantipyrine (AA) and by oxidation to 4-formylaminoantipyrine (FAA). AA is further acetylated by the polymorphic N-acetyltransferase-2 system into 4-acetylaminoantipyrine (AAA). This suggests that certain allergic reactions may be caused by drug metabolites rather than the drug itself, as metamizole is metabolized very rapidly after administration [18].

The analgesic effect of metamizole can be correlated with the concentration of the metabolites MAA and AA, which differ in terms of the onset of effect (MAA > AA) and half-life (MAA: 2.2–3.7 h, AA: 5–8 h). The primary metabolite, MAA, is 50 times more active than metamizole as a COX inhibitor, while the metabolite AA is less active [16].

The half-life of both active metabolites (MAA and AA) is dose-dependent. In healthy individuals, MAA varies between 2.5 h (for 750 mg of metamizole) and 3.5 h (for 3000 mg); for AA, it ranges from 4 to 5.5 h. MAA elimination is prolonged in elderly individuals (t1/2 = 4.5 h) compared to younger subjects (t1/2 = 2.5 h), correlating with creatinine clearance [16].

In humans, patients with liver conditions exhibit a slower elimination of the MAA metabolite compared to healthy patients. In individuals with liver cirrhosis, the plasma half-life of MAA is four times higher than in healthy patients [16].

A study conducted by Bedir [11] found that biochemical experiment results are consistent with histopathological findings. Histopathology indicated that necrosis and mononuclear cell infiltrations developed in hepatocytes in groups treated with 500 and 1000 mg/kg doses. These histological findings were confirmed by the presence of necrosis, apoptosis, fibrosis, and mild portal and lobular hepatitis in the liver tissue of patients treated with metamizole [19].

The effect of metamizole on liver tissue at various doses should be further investigated. It is essential to determine which dose results in the fewest and most dangerous consequences.

According to Malsy et al. [20], metamizole significantly reduced cell proliferation and induced apoptosis in pancreatic cancer cells. Other studies on cell lines have shown that metamizole, alone or in combination with paracetamol, increased apoptosis in colon carcinoma cell lines (SW 480 and HT 29) after 24 and 48 h of exposure [21].

Metamizole also reduced the growth of a human lung cancer cell line in a dose-dependent manner, though it did not affect HeLa cells, as reported by Shao and Feng [22]. On the other hand, Zhang et al. identified a significant neuroprotective effect of metamizole in cerebral ischemia, categorizing it as an anti-apoptotic drug [23].

Several studies have demonstrated that NSAIDs and COX-2 inhibitors reduce proliferation, promote cell death in several cultured cell lines, and enhance the cytotoxic effects of certain antineoplastic drugs through COX-dependent and independent pathways [24]. According to this study [24], the human cancer cells HeLa, HT-29, and MCF-7 showed sensitivity to metamizole after 24 and 48 h. Metamizole caused significant changes in the proliferation and morphology of HeLa cells, with concentrations above 25 µg/mL severely reducing cell viability and proliferation.

This study provides valuable insights into the cytotoxic effects of metamizole and its metabolites, but it has several limitations that should be acknowledged to contextualize its findings and guide future research. This study used the LX-2 liver cell line, which may not fully represent the complexity of liver function and toxicity in vivo. Future studies could incorporate primary hepatocytes or co-cultures of different liver cell types (e.g., hepatocytes, Kupffer cells, and endothelial cells) to more accurately represent the physiological response of the liver to metamizole and its metabolites. Also, this study assessed cell viability and apoptosis after 24 h of exposure. This short exposure period may not capture delayed or long-term toxic effects, which are relevant in clinical scenarios, particularly for drugs that accumulate over time or have cumulative toxicity. By addressing these limitations, future studies can build on these findings to provide a more robust understanding of the hepatotoxic effects of metamizole and its metabolites. To confirm the in vitro findings, in vivo studies are necessary. Animal models could be used to assess liver toxicity following exposure to clinically relevant doses of metamizole and its metabolites. This would help determine whether the observed effects in LX-2 cells are reflective of real-world toxicity and provide a better understanding of potential risks.

Future research should aim to address several key aspects that emerged during this study. The inconsistency in dose ranges between metamizole (100–1000 µg/mL) and its metabolites (100 and 1000 µg/mL) highlights the need for a refined experimental design that considers the potentially higher potency or toxicity of 4-MAA and 4-AA. Determining the precise metabolic profiles and the percentage of each metabolite will allow for more accurate dosage planning, ensuring the relevance of tested concentrations. Expanding this work to include both acute and chronic toxicity studies is also crucial, as most available literature focuses on long-term use, leaving a gap in understanding the interplay between these mechanisms. In vivo studies will provide an opportunity to assess metamizole’s effects in a physiologically relevant setting, offering insights into systemic toxicity and the progression from acute to chronic exposure. Furthermore, investigating the roles of the non-active metabolites 4-FAA and 4-AAA in cytotoxicity, once resources and standard substances become available, will help establish a more complete picture of the metabolic pathways and their potential contributions to adverse effects. These future directions are essential to advancing the translational relevance of our findings and enhancing the safety profile of metamizole.

## 4. Materials and Methods

For each experimental group, cell viability and the degree of apoptosis following exposure to different concentrations of metamizole and its metabolites were determined. The cytotoxic potential of a metamizole solution was tested at 5 concentrations (100, 200, 400, 600, and 1000 µg/mL) and 2 concentrations of the metabolites 4-MAA and 4-AA (100 and 1000 µg/mL).

### 4.1. Chemicals and Reagents

The metamizole solution and the two metabolites were purchased from Sigma-Aldrich (St. Louis, MO, USA). LX-2 cell lines were obtained from MedFuture (Cluj-Napoca, Romania). A Colorimetric Assay Kit was purchased from Elabscience Biotechnology Inc. (Houston, TX, USA) and the Annexin V-FITC kit and propidium iodide (PI) for flow cytometry was purchased from Thermo Fisher Scientific Inc. (Waltham, MA, USA). In this study, we also used a DMEM (high glucose) medium from Sigma-Aldrich; fetal calf serum (Sigma-Aldrich, St. Louis, MO, USA); and 1% antibiotic–antimycotic (Gibco, Miami, FL, USA).

### 4.2. Cell Preparation and Cell Viability

Cells were cultivated in a DMEM (high glucose) medium (Sigma-Aldrich) supplemented with fetal calf serum (Sigma-Aldrich, St. Louis, MO, USA) and 1% antibiotic–antimycotic (Gibco).

The potential cytotoxicity of metamizole and its metabolites was evaluated using an MTT colorimetric assay (3-(4,5-dimethylthiazol-2-yl)-2,5-diphenyltetrazolium bromide). This technique involves the transformation of a yellow MTT compound into purple formazan crystals by enzymes (NAD(P)H-dependent mitochondrial succinate dehydrogenases) that are present in metabolically active cells.

To obtain a single-cell suspension, cells from a confluent culture were treated with a 0.25% trypsin–EDTA solution (Sigma-Aldrich, St. Louis, MO, USA). The obtained cell suspension was centrifuged at 1500 revolutions per minute for 5 min. After quantifying the cells, a concentration of 1 × 10^4^ cells/well was added to 96-well plates in 200 µL of a complete culture medium.

After 24 h, metamizole was added at 5 different concentrations (100, 200, 400, 600, and 1000 µg/mL) and the two metabolites at 2 different concentrations (100 and 1000 µg/mL). The control samples consisted of untreated cells. Each experiment under specific conditions was performed in triplicate. An analysis of cell proliferation was conducted after 24 h.

After 24 h, the medium was removed, and 100 µL of a 1 mg/mL MTT solution (Sigma-Aldrich, St. Louis, MO, USA) was added. After 4 h of incubation at 37 °C in the dark, the MTT solution was removed from each well, and 150 µL of a DMSO (dimethyl sulfoxide) solution (Fluka, Buchs, Switzerland) was added. The optical density of the chromogenic reactions was evaluated using the BioTek Synergy 2 spectrophotometer (Winooski, VT, USA) at a wavelength of 450 nm. Cell viability was calculated considering the optical density of the control culture.

### 4.3. Cell Apoptosis

To evaluate the apoptosis rate in hepatic stellate cells, we used the Annexin V-FITC cellular apoptosis detection kit from Elabscience (Houston, TX, USA). Annexin V is an early marker of apoptosis and is part of the annexin family, which binds to phosphatidylserine (PS), a fluorescent substance. In the presence of a calcium ion (Ca^2+^), Annexin V specifically binds to PS. It is important to note that normal cells do not display phosphatidylserine on the outer layer of a lipid bilayer membrane; it is only expressed on the inner layer. Additionally, Annexin V cannot bind to the cell membrane of necrotic cells. Annexin V-FITC, which is an FITC-conjugated form, selectively binds to PS on the membrane of cells in the apoptotic stage and can be detected using flow cytometry or fluorescence microscopy. Annexin V can be used to monitor cell viability, early apoptosis, late apoptosis, and cell death. This is possible through the use of fluorescent substances such as propidium iodide (PI); 7-amino-actinomycin D (7-AAD); 4′, 6-diamidino-2-phenylindole (DAPI); or PerCP-Cyanine5.5. In the described study, propidium iodide (PI) was chosen for use.

Further, the cells in suspension were treated with a solution of metamizole and its two metabolites. The cells were collected and centrifuged for 5 min, after which the supernatant was removed. To wash the cells, PBS (Phosphate Bovine Serum) was added, and then, they were resuspended and counted. A total of 500 μL of an Annexin V Binding Buffer was added to the cells for resuspension, followed by 5 µL of an Annexin V FITC reagent and 5 µL of a fluorescent substance. After these steps, the samples were homogenized by shaking and were incubated for 15 min at room temperature in the dark. Immediately after the incubation period, the samples were analyzed by flow cytometry using the FACS method (Fluorescence-Activated Cell Sorting). Apoptotic cells were identified as Annexin V-FITC+ and PI−, non-viable cells were identified as Annexin V–FITC+ and PI+, and viable cells were identified as Annexin V–FITC− and PI−.

## 5. Conclusions

Metamizole and its metabolites can significantly reduce cell viability in a dose-dependent manner.

After 24 h of exposure to a metamizole solution, we observed that cell viability decreased progressively with increasing concentrations of metamizole, with a significant decrease at the highest concentration of metamizole, 1000 µg/mL.

The highest decrease in cell viability was noted for both metabolites, 4-AA and 4-MAA, at the 1000 µg/mL dose. For cells treated with this maximum concentration of metamizole, a flow cytometric analysis confirmed the cell viability results, revealing a minimum viability of 54.3%, associated with an early apoptosis rate of 43% and a late apoptosis rate of 2.5%. The flow cytometry results for the two metabolites also demonstrate an increase in apoptotic activity with concentration, showing low apoptosis levels at 100 µg/mL and significantly elevated levels at 1000 µg/mL. Specifically, 4-MAA induced an average of 48% early apoptosis, while 4-AA induced 40.6% early apoptosis at the highest concentration.

These findings suggest that both metamizole and its primary metabolites can induce hepatotoxicity through direct cytotoxic mechanisms on liver cells.

## Data Availability

The data presented in this study are available in the article.
